# Long term effects of aromatase inhibitor treatment in patients with aromatase excess syndrome

**DOI:** 10.3389/fendo.2024.1487884

**Published:** 2024-11-20

**Authors:** Eleni Z. Giannopoulou, Stephanie Brandt, Stefanie Zorn, Christian Denzer, Julia von Schnurbein, Maki Fukami, Alexander Kaiser, Martin Schmidt, Martin Wabitsch

**Affiliations:** ^1^ Division of Pediatric Endocrinology and Diabetes, Department of Pediatrics and Adolescent Medicine, University Medical Center Ulm, Ulm, Germany; ^2^ Center for Rare Endocrine Disease at the University of Ulm, Ulm, Germany; ^3^ German Center for Child and Adolescent Health, partner site Ulm, Ulm, Germany; ^4^ Department of Molecular Endocrinology, National Research Institute for Child Health and Development, Tokyo, Japan; ^5^ Institute for Biochemistry II, Jena University Hospital, Friedrich Schiller University, Jena, Germany

**Keywords:** gynecomastia, estradiol, aromatase, letrozole, testosterone

## Abstract

**Introduction:**

Aromatase excess syndrome (AEXS) is a rare, autosomal dominant disorder, characterized by enhanced aromatization of androgens and estrogen excess. In males it is characterized by pre-/peripubertal gynecomastia, hypogonadotropic hypogonadism, advanced bone age and short adult height. Only a few female patients have been described so far.

**Methods:**

We report on a family with four members with AEXS and present the long-term effects of aromatase inhibitor use in three of them. Genetic analysis showed a monoallelic 0.3-Mb deletion in 15q21, involving parts of *CYP19A1*, *GLDN* and *DMXL2* in all four patients with AEXS.

**Results:**

The index patient (male, 8 years old) presented with gynecomastia and accelerated growth and bone age. With start of puberty, estradiol levels increased, while testosterone levels remained low. Gynecomastia progressed and a mastectomy was performed twice. Presuming AEXS, a therapy with letrozole was initiated at the age of 19 years. Low-dose letrozole treatment was associated with an increase in testicular volume, increase in virilization and improvement in physical strength and libido. His brother (age 3 years) presented with accelerated growth and bone age. Treatment with letrozole, which was started at the age of 7 years, resulted in achieving an adult height of 179 cm and prevented the appearance of gynecomastia. His sister (age 6 years), who presented with premature thelarche and accelerated growth and bone age, was treated with an estrogen receptor modulator and a GnRH analog followed by letrozole treatment. Menarche occurred at age 13.5 years and adult height was 158 cm. Their father had an early, accelerated growth with an adult height of 171 cm, a delayed puberty and no gynecomastia. *In vitro* studies provided evidence for involvement of aromatase induction in atypical cells and an increased range of potential mechanisms regulating aromatase activity due to the presence of the mutated allele.

**Discussion:**

In conclusion, we observed a phenotypic variability within family members with AEXS carrying the same *CYP19A1* microdeletion. When started early, treatment with letrozole was found to prevent the development of gynecomastia and increase adult height in one patient. In adult life, low-dose letrozole treatment resulted in improved physical strength and libido in the index patient.

## Introduction

Aromatase excess syndrome (AEXS, OMIM no. 139300), formerly known as familial gynecomastia, is a rare, autosomal dominant disorder, characterized by enhanced extraglandular aromatization of androgens and estrogen excess (1). AEXS is found to be caused by heterozygous genomic rearrangements in chromosome 15q21.2, causing overexpression of the aromatase gene *CYP19A1* (1, 2). Aromatase is expressed in many tissues and catalyzes the aromatization of the A-ring of androstenedione to produce estrone and the A-ring of testosterone to produce estradiol (3). The prototypical sites of aromatization are the female gonads (ovaries) and the placenta, however, aromatase activity can also be found in other tissues, such as the breast, brain, fetal liver, muscle, bone, testis, skin and adipose tissue, which is the major site of estrogen synthesis in postmenopausal women and in men (4). As so, aromatase is a significant regulator for the balance between estrogens and androgens in both sexes and plays a pivotal role in sexual maturation and pubertal growth.

Around 30 cases of AEXS have been reported so far, the majority of which are male patients (1, 3, 5–12). There is a wide phenotypic variability among patients, which may be due to their genetic finding, including microscopic tandem duplications, microscopic deletions and inversions at 15q21.2 (1–3, 11, 13, 14). The most characteristic clinical feature is bilateral gynecomastia, which typically appears before or during the onset of puberty, mainly due to local conversion of circulating androgens from the adrenal grand (adrenarche) into estrogens (3, 14). The degree of gynecomastia may vary, from mild to severe; however, most of the reported patients underwent a mastectomy at an early age (3, 14). Other clinical features may include accelerated bone age and short adult height due to early fusion of epiphyses (3, 6, 10). In addition, some male patients with AEXS may present with mild follicle stimulation hormone (FSH) dominant hypogonadotropic hypogonadism during puberty, which may inhibit normal testicular growth and virilization (1, 3, 13, 14). Hypogonadotropic hypogonadism may remain in adulthood, however, fertility has been reported to remain unaffected (1, 3, 13, 14). As for female patients, only a few cases have been reported so far, probably due to their milder phenotype or lack of any striking symptoms that would lead these patients to a physician for further investigation (3, 13). In female patients with AEXS, estrogen excess, which is less significant than in males, may lead to premature thelarche, accelerated bone age, short adult height, macromastia, enlarged uterus and/or menstrual irregularities (3, 6, 10, 14). A helpful tool for the clinical diagnosis of AEXS in boys has been previously proposed and includes the following criteria: bilateral and Tanner stage>2 gynecomastia, onset of gynecomastia after the age of 5 years and before the age of 14 years, exclusion of other well-known causes of gynecomastia and having a genetic trait (autosomal dominant) (3). Interestingly, despite the estrogen excess, serum estradiol may be normal in 20% of the patients (3, 12) and some authors suggest using the estradiol/testosterone ratio as index of aromatization (12). A definite diagnosis, though, should be confirmed through genetic testing (3).

In order to decrease the circulating levels of estrogens and, therefore, their action on different tissues, aromatase inhibitors (AIs) are suggested for use in male patients with AEXS (4). AIs were initially developed for the palliative or adjuvant treatment of estrogen-dependent breast cancer as they act by inhibiting the intracellular conversion of androgens to estrogens (4, 15). Up to now, AIs are not approved for any pediatric indication, but are used off-label in children with a few, very rare disorders, as their efficacy is well established. These include AEXS, Peutz-Jeghers syndrome, McCune-Albright syndrome, functional follicular ovarian cysts and testotoxicosis (4, 15, 16). Interestingly, AIs have been tested in boys with idiopathic short stature in order to promote growth, however there are no convincing data to support the beneficial effect of AI therapy on adult height in these boys so far (17). In boys with pubertal gynecomastia, the use of AIs was not found to be effective and is, therefore, not recommended (18). Nowadays the most commonly used AIs in children are the third generation AIs, letrozole and anastrozole. Up to now, there are no pharmacological studies regarding the optimal dose of AIs in children, so children are usually being treated with the same dosage of AIs as adults (15). In male patients with AEXS, AIs are found to increase testosterone levels, promote virilization, increase testicular volume and control the development of gynecomastia (3, 6). A recent study from Binder et al. examined for the first time the effects of a long-term treatment with anastrozole on growth on four male patients with AEXS and showed that AIs may promote adult height when started early (5). There are no reports regarding the use of AIs in females with AEXS so far. In this study, we present the phenotypic characteristics of a family with four members with AEXS, and additionally show the long-term follow-up, from childhood to adulthood, of three AIs-treated patients (two male, one female).

## Materials and methods

### Clinical data

**Figure 1:**
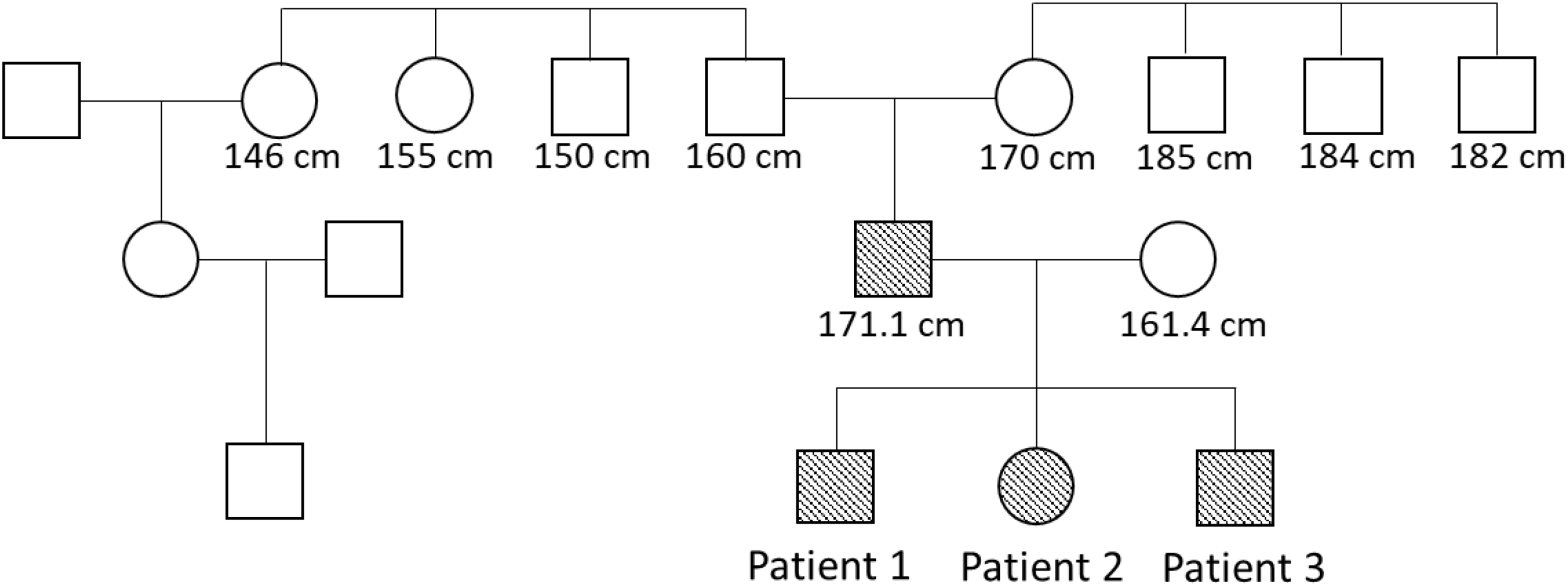
Pedigree. Patients with heterozygous microdeletion in *CYP19A1* are indicated by filled forms. The numbers are adult heights.

We present a family of German origin in which early puberty and short adult height has been reported over three generations in males and females (**Figure 1**). The patients have been reported in a previous study (2), which investigated the genetic basis of AEXS in 6 unrelated patients. The index patient, his parents and his two siblings were examined and treated at the Division of Pediatric Endocrinology and Diabetes, Department of Pediatrics and Adolescent Medicine, University Medical Center in Ulm, Germany. Data from the patients were collected and analyzed retrospectively. In all three children the pubertal stage (development of breast, genitalia and pubic hair) was documented according to the classification by Tanner. Testicular volume was measured using an orchidometer according to Prader (19). Target height was calculated according to Tanner et al. (20). For this study, target height included the height of the affected parent (uncorrected). Height-SDS was calculated using the least mean squares method based on German references (21). Bone age and prognosis of adult height were estimated using the Greulich and Pyle atlas (22) and the Bayley and Pinneau tables for German children (23), respectively.

### DNA sequencing and laboratory data

Leukocyte genomic DNA samples were obtained from the parents and siblings of the index patient. Genomic abnormalities involving *CYP19A1* exons and/or its flanking regions were examined by comparative genomic hybridization (CGH) using a custom-made oligoarray or a catalog human array, as described previously (2).

During treatment with aromatase inhibitor letrozole, assessment of liver function, lipids, hemoglobin and hematocrit were performed every 6-12 months. Hormonal analysis was performed at each appointment and included testosterone, androstenedione, estradiol, DHEA sulfate (DHEAS), luteinizing hormone (LH) and FSH. Hormone serum concentrations were determined with electrochemiluminescent assay (ECLIA). Testosterone was measured by Elecsys Testosterone assay on Cobas pro analyzer (e 801 module) (Roche Diagnostics, Switzerland). The measuring range for testosterone was 0.087−52.0 nmol/L (defined by the Limit of Detection and the maximum of the master curve). Estradiol was measured by Elecsys Estradiol assay on Cobas pro analyzer (e 801 module, measuring range: 18.4−11010 pmol/L) (Roche Diagnostics, Switzerland). Androstenedione was measured by Elecsys Androstenedione assay on Cobas pro analyzer (e 801 module, measuring range: 0.525−34.9 nmol/L) (Roche Diagnostics, Switzerland). LH was measured by Elecsys LH assay on Cobas pro analyzer (e 801 module) (Roche Diagnostics, Switzerland). FSH was measured by Elecsys FSH assay on Cobas pro analyzer (e 801 module) (Roche Diagnostics, Switzerland).

### *In vitro* studies

For the *in vitro* studies, adipose stromal cells (ASC) were isolated from an adipose tissue sample of the index patient during elective surgery (24). Control ASCs were isolated from subcutaneous adipose tissue obtained from reduction surgery. The cells were grown and aromatase activity was measured by the tritium water release assay in 24-well plates using [1β-^3^H]androstenedione (PerkinElmer, Rodgau, Germany) as substrate, as described previously (25, 26). Briefly, incubations with test substances were done for 24 h in cells preconditioned with serum-free medium. Aromatase activities were normalized to the protein content (27). Peripheral blood leukocytes (PBL) were isolated from the index patient and a healthy control using standard procedures. Two million PBL in RPMI1640 medium with fetal calf serum (FCS) were inoculated per well and were directly treated with cortisol or vehicle (ethanol) for 24 h. Values from PBL were normalized to cell number. All conditions were tested in quadruplicate.

From aliquots of ASCs and PBL, total RNA was isolated using the RNeasy Mini Kit (Qiagen, Hilden, Germany) with DNAse digestion. Therefrom, cDNA was synthesized with the High-Capacity cDNA Archive Kit (Applied Biosystems, Darmstadt, Germany) using random hexameric primers. Specific assays based on the Universal Probe Library system (Roche, Mannheim, Germany) were established according to the manufacturer’s standard instructions, as described previously (27). The following assays were used (name, forward primer, reverse primer, probe): full length (exons IX–X), CAA ACC CAA TGA ATT TAC TCT TGA, ACC ATG GCG ATG TAC TTT CC, probe 76; promoter I.1, GTG CTC GGG ATC TTC CAG, CAT GGC TTC AGG CAC GAT, probe 9; promoter I.8, TTG GAC CCC AGA CTT AAG GA, CAT GGC TTC AGG CAC GAT, probe 9; promoter I.4, CAG CCC ATC AAA CCA GGA, CAT GGC TTC AGG CAC GAT, probe 9; promoter I.5, CAG GAT TGA GCA CAC AGG AC, CAT GGC TTC AGG CAC GAT, probe 9; promoter I.7, AGG GGT GAA ATC AGC AAG G, CAT GGC TTC AGG CAC GAT, probe 9; promoter I-f, GAC CAG CAG ACC CAG GAC, CAT GGC TTC AGG CAC GAT, probe 9; promoter I.2, GCT GAT CCC AGT TCT GAA GAG, TCA GAG GGG GCA ATT TAG AG, probe 30; promoter I.6, CAG GAT GTT AGC TGC TCT TCG, CAT GGC TTC AGG CAC GAT, probe 9; promoter I.3, CTT GCC TAA ATG TCT GAT CAC ATT A, CAT GGC TTC AGG CAC GAT, probe 9; promoter II, CCC TTT GAT TTC CAC AGG AC, CAT GGC TTC AGG CAC GAT, probe 9; and GAPDH, AGC CAC ATC GCT CAG ACA C, GCC CAA TAC GAC CAA ATC C, probe 60. All samples were analyzed in duplicate. Relative gene expression was normalized to GAPDH mRNA levels using the comparative cycle threshold method (27).

Statistical evaluation of the *in vitro* experiments was done with 2-sided Student’s t-test (with Bonferroni correction as necessary) as all data sets were normally distributed. Graphs with height trajectories were designed using Graph Pad Prism 7 (Graph Pad Software Inc., San Diego, CA, USA). Percentiles for height were drawn using German reference data (21). Written informed consent was obtained from the patients. The study was approved by the ethics committee of the University of Ulm (247/18) and complies with the declaration of Helsinki.

## Results

### Patient 1 (index patient)

The index patient was primarily seen in our outpatient clinic at the age of 8 years due to bilateral gynecomastia (Tanner stage B2) and accelerated growth (**Table 1**). Physical examination revealed normal prepubertal external genitalia. His bone age was advanced by 5.5 years and the predicted adult height according to the bone age was 159.8 cm, which was significantly lower than his estimated target height (**Table 1**). In the laboratory examination, gonadotropin and testosterone levels were prepubertal and estradiol was undetectable. Hyperthyroidism, hyperprolactinemia, liver disease and renal failure were excluded. Tumors, including testicular tumors (germ cell, Sertoli or Leydig cell tumor) and extragonadal tumors, were excluded by testicular ultrasound and normal hCG, β-hCG and AFP levels in laboratory exams. Urinary steroid excretion analysis revealed normal findings. The patient’s karyotype was 46, XY. Regarding his family history, his father mentioned an early, accelerated growth with a relatively short adult height. The paternal grandfather, two grandaunts and one granduncle reached an adult height of between 150 and 160 cm (**Figure 1**). There was no reported gynecomastia in the family, as well as no infertility problems.

**Table 1:** Clinical characteristics at presentation of patients 1, 2 and 3 with aromatase excess syndrome.

	Patient 1	Patient 2	Patient 3
Sex	Male	female	male
Age (years)	8	6	3
Height (cm) [SDS]	139.9 [+1.7]	126.3 [+1.7]	100.0 [+0.9]
Gynecomastia (Tanner stage)	B2	B3	no
Testes volume (ml)	1	–	1
Pubic hair (Tanner stage)	PH1	PH1	PH1
Bone age (years)	13.5	10.5	5.5
Height prediction (cm) [SDS]	159.8 [-3.1]	146.7 [-3.4]	167 [-2.0]
Target height (cm)* [range: ± 2SD]	172.8 [164.3-181.3]	159.8 [151.3-168.3]	172.8 [164.3-181.3]

SD, standard deviation; SDS, standard deviation scores.

*Target height was calculated according to Tanner and therefore includes the height of the affected parent.

**Figure 2:**
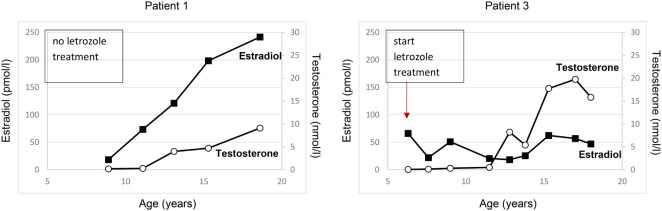
Estradiol and testosterone serum concentrations over time in the two male patients with AEXS. Patient 1 received no aromatase inhibitor treatment till age 19 years. Patient 3 was started on letrozole treatment at the age of 6 years and continued till the age of 18 years. Testosterone levels are shown in white circles, estradiol levels are shown in black squares.

**Figure 3:**
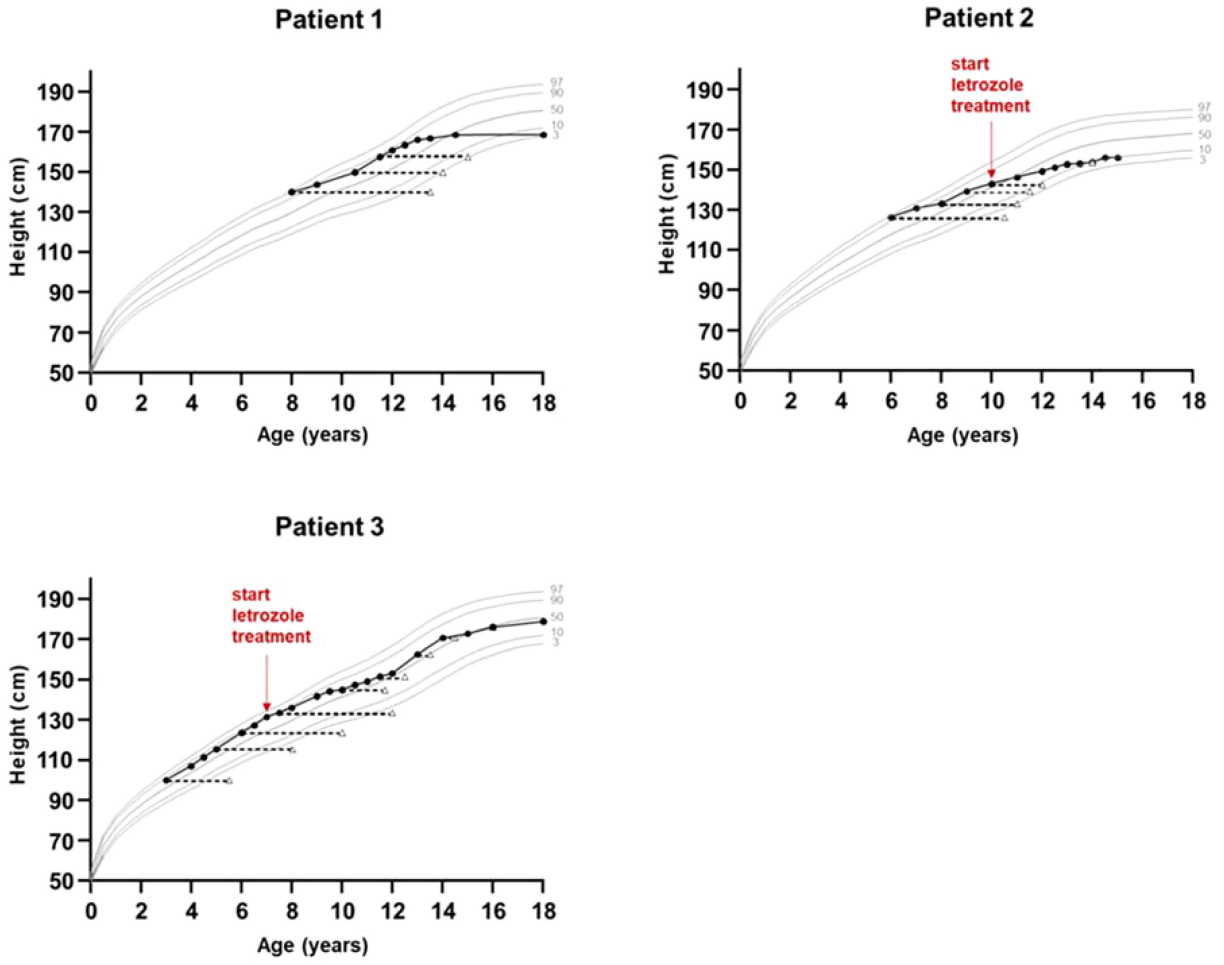
Growth charts of patients with aromatase excess syndrome. Bone age is shown in triangles. Shown are the 3^rd^, 10^th^, 50^th^, 90^th^ and 97^th^ height percentiles according to German reference data for boys (patients 1 and 3) and girls (patient 2).

The index patient was followed regularly in our department for progression of growth, puberty and gynecomastia. Already at the age of 9 ^10/12^ gynecomastia progressed to Tanner stage B4 and due to psychosocial stress, a bilateral mastectomy was performed as a permanent solution. Start of puberty with increase of testicular volume (4 ml) occurred at the age of 11 ^1/12^ years. With start of puberty, gynecomastia progressed again, and in the laboratory examinations serum estradiol levels increased above normal range, whereas testosterone and androstenedione levels remained normal or low (**Figure 2**). At the age of 13 ^2/12^ years a second bilateral mastectomy was performed (Tanner stage B3). Since pubic hair development progressed, there was a slow development of testicular volume, reaching the maximum of 8 ml at the age of 18 years. Regarding his growth pattern, the patient had an early, accelerated growth with growth arrest at age 14 years (adult height: 168 cm, -1.8 SDS) (**Figure 3**).

**Figure 4:**
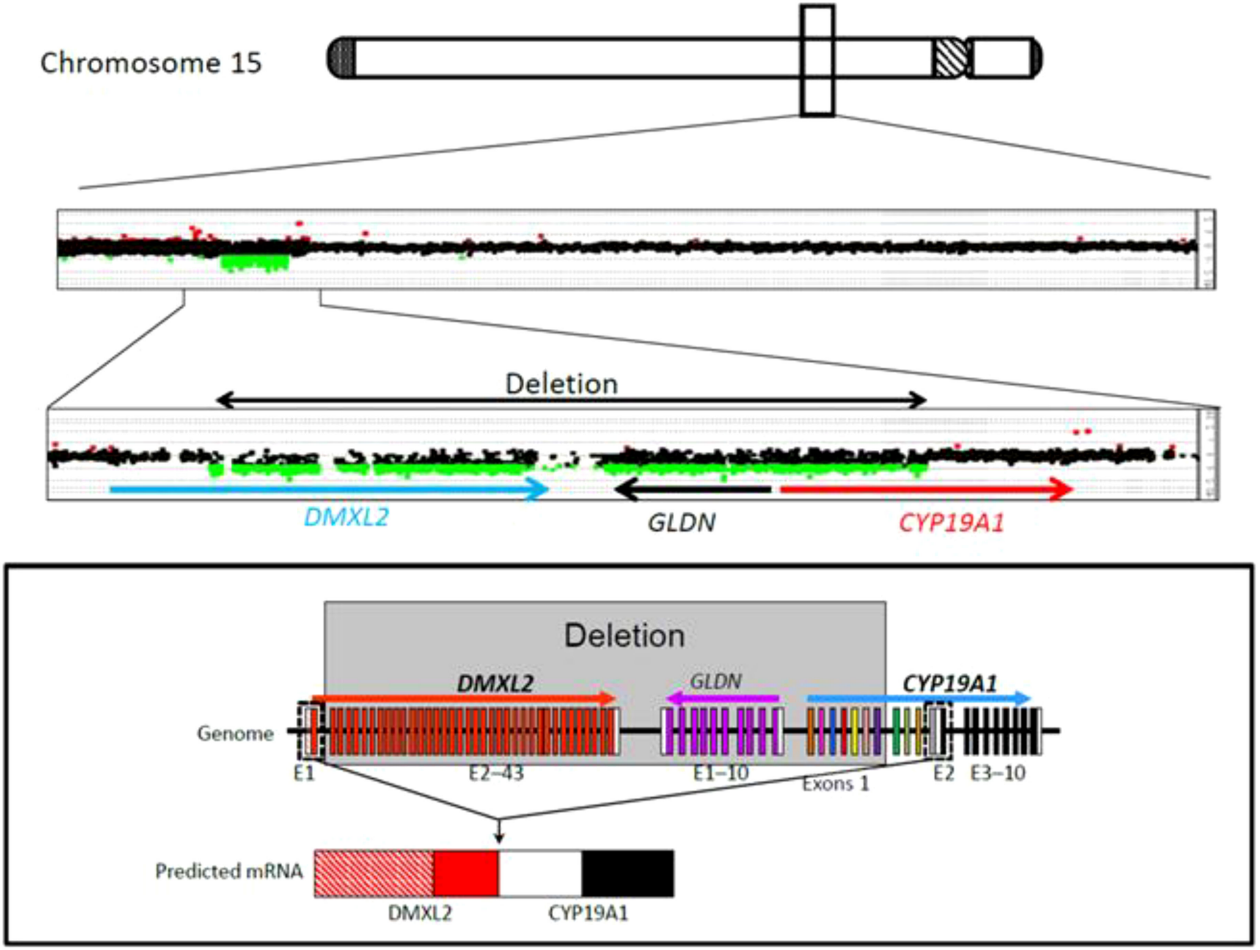
CGH analysis of the four family members with aromatase excess syndrome, revealing a heterozygous microdeletion in the upstream region of CYP19A1.

Presuming an aromatase excess syndrome and while the first genetic findings causing AEXS were published in the literature at that time (1, 10), a genetic analysis of the aromatase gene was performed, which revealed an approximately 0.3-Mb heterozygous deletion in the upstream region of *CYP19A1*. This microdeletion included 7 of the 11 non-coding exons 1 of *CYP19A1*, all exons of *GLDN*, and exons 2-43 of *DMXL2* (2). This deletion is predicted to cause abnormal splicing between *DMXL2* exon 1 and *CYP19A1* coding exons (**Figure 4**). Since DMXL2 is a widely expressed gene (GTEx Portal, https://gtexportal.org/home/), this abnormal splicing likely leads to overexpression of *CYP19A1*. Genetic analysis was consecutively performed to his parents and two siblings; his father, brother and sister were all found to carry the same microdeletion as the index patient (2) (**Figure 4**).

A therapy with letrozole 2.5 mg daily was initiated at the age of 19 years in order to increase testosterone levels and promote testicular volume (7). Testosterone enanthate injections were added to the treatment after 1 year of letrozole therapy because of low testosterone levels and the wish of the patient, due to subnormal masculinization and sparse facial and body hair. Letrozole was stopped and monotherapy with testosterone was continued for 1 year, but there was no change regarding testicular volume and testosterone levels. From the age of 21 years on, the patient was on monotherapy with letrozole, starting again with a full dose of 2.5 mg daily. During treatment testosterone, androstenedione, LH and FSH levels increased gradually, while estradiol levels decreased to normal (**Table 2**). Supraphysiological concentrations of testosterone up to 33.3 nmol/l (reference range: 8.60-29.00 nmol/l) were measured during letrozole treatment. In this case, letrozole dose was gradually reduced and testosterone levels were measured again. When the dose was decreased to 0.1 mg per day, treatment was terminated and the patient was reevaluated after 6 months; interestingly, testosterone levels were found to be low and estradiol levels were elevated, so that treatment with low-dose letrozole was restarted. Letrozole dose was titrated down to a minimum of 0.015 mg/day at the age of 25 years which is still continued (**Table 2**). Low-dose letrozole treatment resulted in gradual promotion of testicular volume (adult volume was achieved at the age of 24 years) and improvement of physical strength and libido. The patient and his wife have one child. No gynecomastia was observed under treatment. Vertebral abnormalities, as assessed by spinal X-rays, were not observed and annual controls of markers of calcium metabolism revealed normal findings. Semen evaluation was refused.

**Table 2:** Laboratory findings before and during treatment with aromatase inhibitor in male patients 1 and 3.

Patient 1					
Age (years)	18	22	25	29	35
Daily aromatase inhibitor dosis	–	2.5 mg	0.02 mg	0.015 mg	0.015 mg
Testosterone (nmol/l)	9.11 (9.70-27.73)	29.82 (9.70-27.73)	17.51 (8.63-29.00)	17.58 (8.63-29.00)	26.41 (8.63-29.00)
Androstenedione (nmol/l)	7.63 (4.50-15.00)	13.23 (2.44-12.57)	5.52 (2.44-12.57)	3.07 (2.44-12.57)	3.11 (0.98-5.31)
Estradiol (pmol/l)	241.6 (27.90-156.40)	23.50 (27.90-156.40)	70.49 (27.90-156.40)	83.71 (27.90-156.40)	158.60 (27.90-156.40)
LH (mIU/ml)	2.07	44.80	4.50	N/A	N/A
FSH (mIU/ml)	1.20	24.90	3.00	N/A	N/A
Patient 3					
Age (years)	6	8	15	18	28
Daily aromatase inhibitor dose	–	2.5 mg	0.6mg	0.3 mg	–
Testosterone (nmol/l)	<0.09 (0.13-1.01)	0.14 (5.89-29.47)	17.75 (9.70-27.73)	15.81 (9.70-27.73)	4.47 (8.63-29.00)
Androstenedione (nmol/l)	N/A	N/A	9.36 (2.44-12.57)	8.66 (4.50-15.00)	1.60 (0.98-5.31)
Estradiol (pmol/l)	66.08 (<73.40)	22.00 (<73.40)	62.40 (27.90-156.40)	47.00 (27.90-156.40)	175.50 (27.90-156.40)
LH (mIU/ml)	<0.10	0.40	2.90	2.18	6.65
FSH (mIU/ml)	0.67	2.21	5.95	6.71	3.94

LH, luteinizing hormone; FSH, follicle stimulating hormone.

### Patient 2 (sister of index patient)

The sister of the index patient presented at the age of 6 years with premature thelarche (Tanner stage B3), accelerated growth and advanced bone age of 10 ^6/12^ years (**Table 1**). At this time point the adult height prognosis was estimated to be 146.7 cm (range ±2SD: 140.9-152.5 cm) (23), which was significantly lower than her estimated target height (159.8 cm, range ±2SD: 151.3-168.3 cm). Laboratory examinations revealed low gonadotropin levels and a serum estradiol level of 77 pmol/l. A gonadotropin-releasing hormone stimulation test excluded central precocious puberty, and an off-label therapy with tamoxifen, an estrogen receptor antagonist, was initiated at the age of 7 years due to precocious thelarche and growth acceleration. At the age of almost 10 years and because of vaginal bleeding, the patient was started on a GnRH analog. At the age of 11 years and after receiving the genetic diagnosis of AEXS, 2.5 mg letrozole daily were added to the therapy. At that time her height was 146.3 cm and her bone age was 12 years (**Figure 3**). At the age of 12 years treatment with both drugs was stopped and menarche occurred at age 13 ^6/12^ years. The patient reached an adult height of 158 cm (-1.6 SDS) (21), which was within her estimated target height range (mean target height: 159.8 cm, range ±2SD: 151.3-168.3). Pelvic ultrasound examinations revealed no pathological findings, and following the next years no menstrual irregularities were reported till the age of 16 years, when the use of a birth control pill was initiated due to her wish for contraception. All markers of calcium metabolism are up to present within normal range.

### Patient 3 (brother of index patient)

A younger brother of the index patient presented at the age of 2 ^11/12^ years with accelerated growth and bone age (Tanner stage B1, PH1, bone age 5 ^6/12^ years) (**Table 1**). Following the diagnosis of his brother, a genetic analysis was performed and confirmed AEXS. In cognition of his brother’s history, treatment with 1.25 mg letrozole daily was started at the age of 6 ^4/12^ years, in order to control growth, sexual maturation and the development of gynecomastia. At that time his height was 127.3 cm and his bone age was 10 years (**Figure 3**). The dose of letrozole was soon increased to 2.5 mg per day; serum levels of gonadotropins, testosterone and estradiol were regularly screened during treatment (**Table 2**). Letrozole treatment during puberty resulted in hormonal balance between estradiol and testosterone (**Figure 2**). Puberty started at the age of 10 years and testicular volume developed normally. Due to elevated testosterone levels from the age of 12.5 years, the dose of letrozole was gradually decreased reaching the minimum of 0.3 mg per day at age 16 years. He continued with this dose till the age of 18 years. Due to the patient’s wish a bilateral, prophylactic mastectomy was performed and treatment with letrozole was stopped thereafter. The patient reached an adult height of 178.8 cm (target height: 172.8 cm, range ±2SD: 164.3-181.3 cm). Virilization and adult testicular volume were reached at age 16 years. Gynecomastia was not observed. We did not observe any side effects during treatment with letrozole. Markers of calcium metabolism in blood remained within normal range. Semen evaluation was refused.

### Patient 4 (father of index patient)

The index patient´s father had an early, accelerated growth with a relatively short adult height (**Figure 1**), which was, however, within his estimated target height. He mentioned a delayed puberty and his first shave was at the age of 17 years. During his whole life he mentioned sparse body and facial hair. He had no gynecomastia. Laboratory examination revealed elevated estradiol and low testosterone levels (data not shown).

### Results from *in vitro* studies

**Figure 5:**
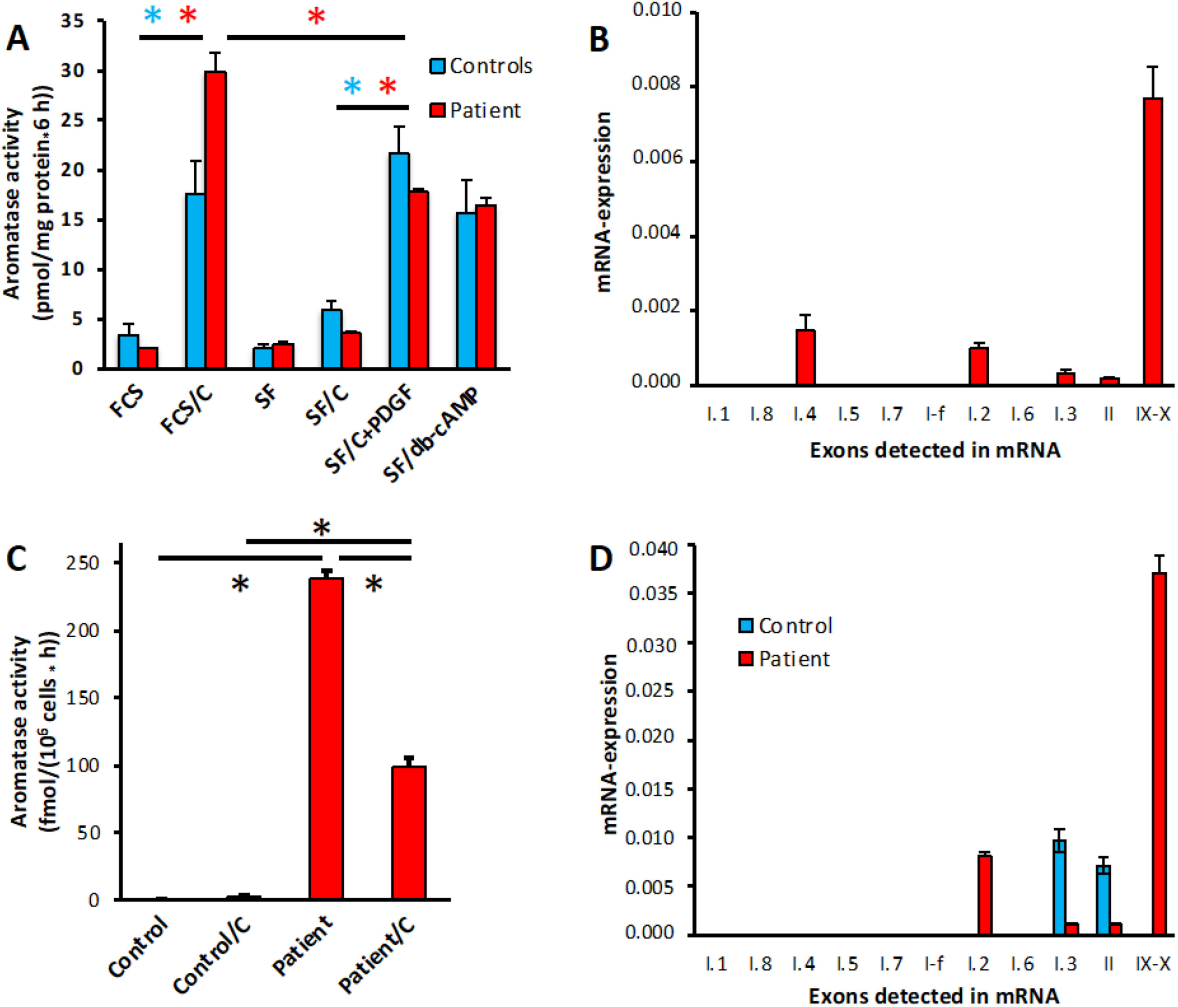
Aromatase activity and promoter usage in ASCs and PBL. **(A)** Aromatase activity in patients ASCs treated in the presence (FCS) or absence (SF) of serum with vehicles or the indicated inducers: 1 µM cortisol **(C)**, 0.5 nM PDGF-BB (PDGF) or 1 mM dibutyryl-cAMP (db-cAMP). Normal control data were taken from a matched group of donors (n=12) undergoing elective adipose tissue reduction surgery, which were cultured under the same conditions as the patient’s cells. **(B)** Promoter usage in patient ASCs. Promoter specific mRNAs are detected by qPCR and are indicated in their normal order on the DNA, IX-X indicates full-length transcripts. Expression is normalized to *GAPDH*. **(C)** Aromatase activity in PBL from a unaffected donor (control) or the patient, treated with vehicle (ethanol) or 1 µM cortisol **(C)** for 24 h. **(D)** Promoter usage in PBL from the same donors as used in **(C)**. Aromatase activities were assayed in quadruplicate replicates, qPCR was done in duplicates. *, p<0.001.

To further verify manifestations of aromatase excess resulting from the microdeletion found via CGH, we analyzed aromatase activity and mRNA-expression in ASCs, which normally express significant amounts of aromatase, and in PBL, which normally show only marginal signs of aromatase expression. The pattern of aromatase activities resulting from various (combinations of) inducers in ASCs from the index patient appeared almost normal, as compared with a group of 12 controls matched for equal duration of cultivation of the ASCs (**Figure 5A**). It revealed the typical induction by cortisol in the presence of FCS, which in the absence of serum can be replaced by a growth-factor like platelet-derived growth factor (PDGF), and which activates promoter I.4. In normal ASCs, the activities induced thereby are roughly equal (28). However, in the patient´s cells the induction in the presence of FCS was significantly stronger, suggesting some additional induction mediated by another pathway. Analysis of the appearance of the typical alternative, untranslated first exons in the aromatase mRNA allows determination of the promoters used for induction. It revealed the appearance of exon I.2-containing mRNAs, in addition to mRNAs containing exons I.4, I.3 and II in normal ASCs (**Figure 5B**), thus confirming the CGH-results.

Aromatase activity (**Figure 5C**) and expression of full-length mRNA containing exons IX and X (**Figure 5D**) are barely detectable in normal PBL (where appearance of exons I.3 and II indicates the expression of some truncated transcripts). In contrast, the patient’s PBL exhibited significant aromatase activity and full-length mRNA, driven mostly by exon I.2 containing transcripts originating from the allele with the microdeletion. Interestingly, in these PBL cortisol decreased aromatase activity, which might be attributed to cortisol acting on the *DMXL2* promoter.

## Discussion/conclusion

AEXS is a rare disorder characterized by enhanced extraglandular aromatization of androgens and estrogen excess (1). In order to increase adult height, promote testicular development and prevent gynecomastia, 3^rd^ generation AIs are used off-label in male patients with AEXS (3, 5). Here, we presented a family with four members with AEXS, in which the three offspring were treated with the 3^rd^ generation AI, letrozole. The 8 year old index patient, who presented with gynecomastia, tall stature and accelerated bone age, was started on letrozole treatment at the age of 19 years, which resulted in sexual maturation. The brother of the index patient presented at the age of 3 years with accelerated growth and bone age. Letrozole treatment was started at the age of 6 years and resulted in achieving an adult height within his estimated target height range, prevention from the development of gynecomastia, and promotion of sexual maturation. The sister of the index patient, who had a history of premature thelarche, accelerated growth and bone age, was treated at first with an estrogen receptor antagonist followed by a treatment with letrozole and GnRH analog and resulted in reaching an adult height within her estimated target height range.

Prepubertal gynecomastia is not a common finding in young boys. AEXS is a rare cause of prepubertal, bilateral gynecomastia in boys, occurring during adrenarche in most of the reported cases with AEXS (3). The differential diagnosis of gynecomastia mainly includes secondary causes like chromosomal anomalies (Klinefelter syndrome), enzyme deficiencies (e.g. 17β-hydroxysteroid dehydrogenase deficiency, 21-hydroxysteroid dehydrogenase deficiency, 17α-hydroxylase deficiency syndrome deficiency etc.), liver cirrhosis, tumors (e.g. hCG-producing tumors, choriocarcinoma, germ cell tumors, estrogen-producing tumors etc.), renal disease and use of specific drugs (e.g. aldosterone receptor blockers, anti-hypertensive drugs, psychotropic drugs etc.) (3). Physiological pubertal gynecomastia is a common condition that usually occurs around the age of 14 years in otherwise healthy boys, has a slow progression and resolves spontaneously within 24 months (3, 18). Accelerated growth, advanced bone age, short adult height, underdeveloped testes during puberty and sparse body and facial hair are suggestive of estrogen excess and should provoke further laboratory and genetic testing. As in our case, a positive family history including cases of gynecomastia and short adult height, is definitely a strong indicator for further investigation.

Gynecomastia in young male individuals may have a significant impact on their self-esteem and quality of life. The majority of reported male cases has undergone mastectomy as early as the age of 12 years (3, 5). Although rare, there are cases of recurrence of gynecomastia after mastectomy in patients with AEXS (5). In the present study, prophylactic and early letrozole use prevented the appearance of gynecomastia during puberty in one patient. Binder et al. showed, similarly, that gynecomastia regressed within a few months after start of treatment with a third generation non-steroidal AI, anastrozole, in 4 boys with AEXS (5). Regarding recurrence of gynecomastia, the authors suggested timely mammoplasty after cessation of treatment with anastrozole (5). In our study, the brother of the index patient underwent a prophylactic mastectomy after cessation of AI treatment; the patient has not developed gynecomastia already ten years after end of treatment with letrozole. A close follow-up is suggested, however, in these patients. Patients with an early diagnosis of AEXS, preferably before onset of puberty, may benefit from a prophylactic treatment with AIs, not only regarding gynecomastia, but also regarding growth, as explained below.

Apart from the positive effects on the development of gynecomastia, the use of AIs in male patients with AEXS has been found to improve adult height, promote virilization and increase testicular volume (5, 7, 10, 12, 29). So far, anastrozole has been used in the majority of studies with patients with AEXS, while letrozole has been used in combination with growth hormone treatment in only one study (12). Both anastrozole and letrozole suppress estrogen production by 97-99% and are highly selective (4). In the current report, letrozole was selected due to its higher potency in inhibiting aromatase activity. Letrozole is, like anastrozole, rapidly absorbed, but has a longer half-life of 2 to 4 days, leading to higher plasma testosterone concentrations (4, 30). Indeed, supraphysiological levels of testosterone were observed during letrozole treatment in the two male patients in the present study. Because of increased testosterone levels, the dose of letrozole has been titrated down to a minimum of 0.015 mg per day in the index patient and 0.3 mg per day in the brother of the index patient. As of note, the index patient was started on letrozole in adulthood, while his brother in childhood. The increase in testosterone levels was accompanied by a concomitant rise in LH and FSH concentrations; clinically an increase in testicular volume was observed. Our findings are supported by previous research showing also a supraphysiological rise in testosterone concentrations and rapid testicular growth due to increase in gonadotropin secretion in boys with idiopathic short stature treated with letrozole (30). Suppression of estrogen biosynthesis by letrozole decreases the negative feedback control of gonadotropin secretion and raises concentrations of serum FSH and LH (30). Regular follow-up of these hormones is suggested and appropriate adjustments of letrozole dose should be considered during letrozole treatment.

Another significant effect of early administration of AIs in patients with AEXS is on adult height (5). Estrogen excess already before the onset of puberty accelerates skeletal maturation and growth and causes premature closure of the epiphyses (3, 5). Adult heights of untreated patients with AEXS are found to range between -2.5 and 0 standard deviations of normal (3). So far, there is only one study that has investigated the long-term effect of AIs (anastrozole) on adult height in patients with AEXS (5). This study showed that early initiation of 1.0 mg anastrozole therapy resulted in improved adult height in two male patients, compared with the initial prediction (+6.9 and +8.1 cm) (5). The results of our current study are in agreement with previous findings, as the younger brother, who was started early on AI treatment reached his estimated target height and improved his initial prediction (+8.4 cm). Of note, this is the youngest reported male patient with AEXS treated with AIs, compared to previous studies (5). Adult height of patients with AEXS should be carefully assessed in terms of uncorrected target height, especially if one of the parents is affected with AEXS, as in the present study. While the two patients in this study reached their genetic potential based on midparental height, midparental height may underrepresent true target height in case the father’s height was compromised due to AEXS. Still, early intervention, when possible before puberty onset, should be considered in patients with AEXS in order to improve adult height.

The female patient in the present study reached an adult height within her estimated target height range. However, her course was complicated by the presence of precocious puberty, as well as by the administration of an estrogen receptor antagonist and a GnRH analog before AI treatment. Therefore, although her height prognosis was improved, a clear conclusion about the effect of letrozole use on height development cannot be drawn. Regarding females with AEXS, the main challenge remains the diagnosis, especially when it comes to sporadic cases, when there are no affected male members in the same family. In the literature there are only a few reports on female patients with AEXS describing their phenotype, which includes premature thelarche, accelerated bone age, short adult height, macromastia, enlarged uterus and/or menstrual irregularities (3, 6, 10, 14). There is no available information regarding AI treatment, mainly due to concerns that treatment with AIs may cause ovarian overstimulation in females (31). Since a positive result of AI treatment on adult height has been proved in males, a short-term AI use should be considered in females with AEXS in order to improve adult height.

In AEXS, phenotypic severity seems to be determined by the expression levels of *CYP19A1* due to the type of genomic rearrangement (1–3, 11, 13, 14). As such, patients with inversions are found to show an early disease onset with severe gynecomastia, advanced bone age and short adult height, while patients with duplications show mild gynecomastia with pubertal onset and normal adult height. Patients with deletions are found to exhibit an intermediate phenotype (2, 3, 13). Interestingly, in the present study we observed a phenotypic variety within family members carrying the same microdeletion of *CYP19A1*. This finding needs to be confirmed also in other family studies in order to identify the factors responsible for this phenotypic variety.

Our study also verified atypical expression patterns of *CYP19A1*/aromatase in affected patients. Analysis of aromatase activity and mRNA-expression in ASCs, which normally express significant amounts of aromatase, revealed a potentiation of inducible activity in the presence of the mutated allele when cortisol and FCS were used for induction. However, due to the young age of the index patient at the time of surgery it was not possible to obtain significant numbers of age matched controls. Therefore, matching was based on equal duration of cultivation of the ASCs, as there is a significant influence of duration of *in vitro* cultivation on aromatase activity of ASCs (32). Despite this limitation of the experiments with ASCs, it is obvious that the mutated allele is linked to an altered pattern of aromatase activities in these cells. Furthermore, and perhaps more important, we showed that the mutated allele is sufficient for induction of functional aromatase expression in cells not normally doing so. Our results further indicate that also in cells with ectopic expression of aromatase its activity may be regulated by hormonal factors like cortisol, which obviously does not always act as an inducer of aromatase in these patients’ cells/tissues. This may at least in part explain the large phenotypic variety seen in AEXS patients.

In most of the previous reports, AI use was terminated when adult height was reached (5). A long-term use of AIs extending to adult life in patients with AEXS has been previously suggested (3), however studies are lacking. In the present study we examined the use of low-dose letrozole during adult age in one patient. During adult age, AI use at very low doses resulted in normal serum levels of estrogens and androgens. Clinically, AI treatment improved physical strength and libido. A long-term use of AIs at a low dose should be considered in adult male patients with AEXS. It is important, though, that more cases are documented. The consequences of long-term hyperestrogenemia in patients with AEXS regarding coronary heart disease as well as breast and prostate cancer are unknown, so a regular follow-up for these conditions is suggested.

Markers of bone health (calcium metabolism) in our two long-treated patients showed no pathological findings. In addition, no history of bone fractures was reported. Patients with AEXS are expected to have normal or even increased bone mineral density (5, 7). During treatment with AIs, bone health is not expected to be impaired as estrogen levels are regularly screened and are aimed to remain at normal levels. Accordingly, Binder at al. showed that treatment with anastrozole did not reduce bone density as controlled by DEXA scan in two patients (5). No detrimental effects of AI use on bone health have been reported in males so far (3).

Limitations of the present study is the limited number of included patients and the absence of a control group. However, AEXS is an extremely rare disorder with only a few reported cases so far, therefore our study adds new knowledge to the current literature.

In conclusion, the present study provided information on the clinical phenotypes of four family members with AEXS as well as atypical expression patterns in ASCs and PBL. Long-term low dose letrozole treatment in adulthood promoted testicular growth and improved physical strength in one adult patient. In the second male patient, initiation of letrozole treatment before onset of puberty resulted in improved adult height and prevented from the appearance of gynecomastia. The female patient, who had a history of precocious puberty, received a combined treatment including letrozole, which resulted in reaching her estimated target height. While AEXS is a rare disorder, diagnosis should be suspected in cases of prepubertal gynecomastia, accelerated growth and advanced bone age, and confirmed through genetic testing. More reports with long-term follow-up data are still needed in order to prove the positive effects of AI treatment on adult height and gynecomastia.

## Data Availability

The datasets presented in this study can be found in online repositories. The names of the repository/repositories and accession number(s) can be found in the article/supplementary material.
